# Clinical Profile of Optic Neuritis in Malaysian Patients Older Than 45 Years of Age

**DOI:** 10.7759/cureus.23571

**Published:** 2022-03-28

**Authors:** Mohammad Hudzaifah-Nordin, Chin Feng Wendy-Ong, Masnon Nurul-Ain, Wan-Hazabbah Wan Hitam

**Affiliations:** 1 Department of Ophthalmology and Visual Sciences, Universiti Sains Malaysia School of Medical Sciences, Kelantan, MYS; 2 Faculty of Medicine, Universiti Sultan Zainal Abidin, Terengganu, MYS; 3 Department of Ophthalmology, Hospital Kuala Lumpur, Kuala Lumpur, MYS

**Keywords:** visual acuity, papilledema, optic neuritis, middle-aged, aged

## Abstract

Introduction

Optic neuritis (ON) is a blinding inflammatory disease of the optic nerve, typically affecting young adults as described in the Optic Neuritis Treatment Trial (ONTT). However, there is limited information describing ON in patients older than 45 years of age. The aim of this study was to determine the clinical profile of ON in this age group in Malaysia.

Methods

A retrospective case series study was performed between January 2014 and December 2018 among patients older than 45 years old, treated as ON in Hospital Universiti Sains Malaysia, Kelantan, Malaysia. Clinical features, visual acuity, imaging, laboratory results, aetiologies, and visual outcomes were analyzed.

Results

This study comprised 17 patients (20 eyes) with a gender distribution of eight males and nine females. The mean age of ON onset was 55.8 ± 7.7 years (46-80 years). Most patients (94.1%) presented with reduced vision and retroorbital pain, and three patients (17.6%) had bilateral eye involvement. Papillitis was observed in 13 eyes (65%), retrobulbar ON in five eyes (25%), and optic atrophy in two eyes. Presenting visual acuity was moderately correlated with final visual outcome (rs= 0.47, n = 20, p < 0.05). Post-treatment visual improvement (good and slight) was reported in 14 eyes (70%), four (20%) had no improvement while the remaining two eyes (10%) had worsening best-corrected visual acuity (BCVA). Infective causes accounted for 23.5% of cases (one ocular syphilis, two ocular tuberculosis, and one case of toxoplasmosis). Most cases (70.6%) were idiopathic ON, and only one case was diagnosed with neuromyelitis optica spectrum disorder (NMOSD).

Conclusion

ON in Malaysian patients older than 45 years of age has no gender predilection and is most likely to be idiopathic; however, infective ON must still be ruled out. Overall visual outcomes were satisfactory. Among the infective causes, ocular tuberculosis caused poor visual recovery as compared with syphilitic and toxoplasmosis-related ON cases.

## Introduction

Optic neuritis (ON) is an acute inflammatory optic nerve disease that can be either typical or atypical based on its clinical presentation and natural history. Demyelinating inflammatory diseases are the most common cause of ON, which is usually associated with multiple sclerosis (MS) and neuromyelitis optica (NMO) or can occur in isolated episodes [1]. MS is more common in Western countries, whereas NMO has a higher prevalence in Asia [2]. Infection is also another important etiology of ON that needs to be considered [3].

The Optic Neuritis Treatment Trial (ONTT) reported that the most commonly affected age range is 18 to 45 years old, with the mean patient age being 31.8 years old [4]. ON clinical profiling in the Malay population revealed a peak age among young adults between 21 and 30 years of age; the elderly age group, although included in the study sample, was not further discussed in detail [5]. There are limited studies describing ON features in populations older than 45 years of age. This can be due to the rarity of ON, and this is oftentimes misdiagnosed as non-arteritic anterior ischaemic optic neuropathy (NAION) due to the overlapping clinical profiles of these two diseases [6]. There are also inconsistent findings describing ON in middle-aged and elderly populations in China, Korea, and the US [2,6-8]. Therefore, to improve the understanding of this disease in our population, we described the clinical manifestations, visual outcomes, and etiology of ON in patients in this age group.

Part of the manuscript has been presented as an oral presentation at the 10th Asian Neuro-Ophthalmology Society Congress on March 3, 2019, in Manila, Philippines.

## Materials and methods

Method

A retrospective case series study was performed on 17 patients who were treated for ON at Hospital Universiti Sains Malaysia, Kota Bharu, Malaysia, between January 2014 and December 2018. The study was conducted according to the Declaration of Helsinki and was approved by the Research and Ethical Committee of the School of Medical Sciences, Universiti Sains Malaysia.

Inclusion criteria

Patients aged older than 45 years old and diagnosed with ON were included in this study. These patients were diagnosed clinically with their acute presentation of a decrease in visual acuity or visual field, with or without eye pain. Other diagnostic criteria included positive relative afferent pupillary defect (RAPD), reduced color vision, reduced light brightness sensitivity, nerve fiber bundle visual field defect, and optic disc swelling or atrophy.

Exclusion criteria

Exclusion criteria included other causes of optic neuropathy, such as NAION, and compressive, traumatic, infiltrative, hereditary, or glaucomatous optic neuropathy.

Data collection

We reviewed the age of onset, gender, initial presenting ocular complaints, and associated symptoms. Full ophthalmic examinations performed were evaluated, including best-corrected visual activity (BCVA), pupillary examination, optic nerve functions, color vision and light brightness, fundus examination, and visual field assessment. Imaging studies completed to identify optic nerve pathology or abnormal enhancement and other brain pathology were also reviewed. We also reviewed infective screening for tuberculosis, syphilis, and toxoplasmosis, which had been performed in certain patients. Inflammatory and autoimmune studies, such as serum antinuclear antibody (ANA), anti-double-stranded DNA (anti-dsDNA), rheumatoid factor, and aquaporin-4 antibody, were also studied. Considering the retrospective nature of this study, radiological and blood examinations could not be evaluated in several patients. The mode of treatment and visual outcomes were also analyzed.

Statistical analysis

All analyses, which were mostly descriptive analytics, were performed using IBM SPSS Statistics for Windows, Version 24.0. (IBM Corp. released 2016, Armonk, NY).

## Results

A total of 17 patients (20 eyes) were included in this study, of which the gender distribution was nearly equal (8 males and 9 females). The mean age of onset was 55.8 ± 7.7 years (46-80 years). The sample included 15 (88.2%) Malay patients, and the remaining patients were of Chinese ethnicity.

During the initial presentation, only one patient (5.9%) presented with painless reduced vision while the other patients reported retroorbital pain. Bilateral involvement was observed in three patients (17.6%). Papillitis was reported in 13 eyes (65.0%), five eyes (25.0%) showed evidence of retrobulbar ON, and two eyes had optic atrophy. No cases of neuroretinitis were noted (Table 1). All unilateral ON patients had RAPD in the affected eye, while bilateral ON patients had RAPD in the more severely affected eye.

**Table 1:** Demographic and clinical characteristics of ON patients, laboratory and neuroimaging results, and treatment received. a) Based on 20 eyes b) Rheumatoid factor ON: optic neuritis; NMOSD: neuromyelitis optica spectrum disorder

Variables	Number of patients, n=17 (%)
Gender	
Male	8 (47.1)
Female	9 (52.9)
Age of onset (years)	
Mean	55.8 ± 7.7 (range 46-80)
45–54	8 (47.1)
55–64	8 (47.1)
65 and above	1 (5.9)
Race	
Malay	15 (88.2)
Chinese	2 (11.8)
Ocular pain	16 (94.1)
Laterality	
Unilateral	14 (82.4)
Bilateral	3 (17.6)
Optic disc^a^	
Papillitis	13 (65.0)
Retrobulbar ON	5 (25.0)
Atrophy	2 (10.0)
Neuroretinitis	0 (0.0)
Imaging	
Normal	3 (17.6)
Abnormal	7 (41.2)
Not completed	7 (41.2)
Positive Mantoux test	2 (11.8)
Positive Toxoplasma IgM/IgG	1 (5.9)
Positive aquaporin-4 antibody	1 (5.9)
Positive VDRL	1 (5.9)
Positive ANA	0 (0.0)
Positive anti-dsDNA	0 (0.0)
Positive RF^b^	0 (0.0)
IV corticosteroid therapy	
Yes	13 (76.5)
No	4 (23.5)
Relapse	2 (11.8)
Final diagnosis	
Idiopathic ON	12 (70.6)
Infective ON	4 (23.5)
NMOSD	1 (5.9)

The mean presenting BCVA was 3/60, with 10 of the affected eyes (50%) having a BCVA of 6/60 or worse. The presenting BCVA was moderately correlated with final visual outcome (rs = 0.47, n = 20, p < 0.05). Good visual improvement (i.e., ‘good’ and ‘slight’ improvement) was seen in 70% of the involved eyes, the status of the other 20% remained stable, and the remaining eyes were noted to have worsening visual acuity during the patients’ final visits (Table 2). The proportion of patients with a BCVA of 6/60 or worse decreased to 30% post-treatment while 50% of the affected eyes were reported to have an outcome BCVA of 6/12 or better during the final review.

**Table 2:** BCVA of ON patients at initial presentation and final post-treatment examination ON: optic neuritis; BCVA: best-corrected visual acuity

Variables	Initial presentation	Final examination
	n=20 eyes (%)	n=20 eyes (%)
6/6 or better	0 (0)	2 (10.0)
6/7.5 to 6/12	4 (20.0)	8 (40.0)
6/15 to 6/48	6 (30.0)	4 (20.0)
6/60 or worse	10 (50.0)	6 (30.0)

Imaging examinations of the brain and orbit were performed in 10 patients: three patients had optic nerve enhancement and swelling on MRI, one had T2 lesions, and three patients had ischemic foci. An investigation of one patient revealed aquaporin-4 antibody seropositivity.

Treatment involved intravenous methylprednisolone therapy for three days followed by oral prednisolone after infective causes had been ruled out in the initial phase for 13 patients; four patients were treated accordingly for infective causes. During the final follow-up, 12 patients (70.6%) were diagnosed with idiopathic ON, one patient (5.9%) with NMOSD, and the remaining four patients (23.5%) with infective ON (2 cases of ocular tuberculosis, 1 case of ocular syphilis, and 1 case of toxoplasmosis). The average duration of follow-up for our patients was two years and eight months. Multiple relapses were noted for two patients (11.7%) throughout the follow-up period.

## Discussion

There is a paucity of information describing the clinical profile of ON in middle-aged and elderly populations. To our knowledge, only three articles from China [2,7] and Korea [6] have described ON profiles in this age group in the Asian region. The ONTT and a few other studies reported female predominance among ON patients [2,4,9]; although the background of this observation is unclear, it was believed to be due to the twofold risk of developing a demyelinating disease (MS) in young women [10]. In our study, however, there was no observed gender predilection; this was probably due to only a single case of demyelinating disease (NMOSD) in our sample population. The mean age of onset was 55.8 ± 7.7 years, which is comparable to studies by Wang et al. (55.5 ± 8.29 years) [2]. The majority of our patients were Malay (88.2%) and the remaining were of Chinese ethnicity, which was comparable to previous work by Shatriah et al. at our center, revealing that most ON cases occurred in Malays [5].

The presenting feature of retroorbital pain was reported in 94.1% of our patients, which was consistent with the ONTT prevalence of 92.2% [4]. We could not further classify the severity of the pain due to the retrospective nature of this study. Most of our patients presented with unilateral eye involvement, in parallel with an Asian observational cohort study [11] and the Chinese elderly population [2]. The frequency of papillitis was noted to be higher than retrobulbar optic neuritis among our patients. This finding was consistent with a previous study by Shatriah et al. [5] and studies in other Asian countries [11-12]; however, it was in contrast with the ONTT, which recorded higher retrobulbar ON in Western populations. Woung et al. suggested that this finding was consistent with the low prevalence of MS among Asians, as papillitis patients have a low risk of developing MS [10].

From our review, we found a significant association between the initial and outcome BCVA; patients with poor visual outcomes were those who had poorer presenting BCVA (Table 3). The ONTT and Wang et al. also reported similar findings [2,4]. Two recent studies from China reported that the initial BCVA for ON patients was better in the younger age group [2,7]. We found a similar correlation between younger presenting age and better visual acuity; however, this correlation was weak and not statistically significant. We observed good visual improvement among our patients’ age group (Table 4), similar to the findings of Choi et al. [6]. Visual improvement was noted in 70% of the eyes (10 eyes with good improvement and 4 eyes with slight improvement). In addition, BCVA at initial presentation in 50% of patients was 6/60 or worse, and BCVA improved to 30% at the final examination. This finding is in parallel with the good visual recovery observed in younger patients in the ONTT and in a previous study at our center on ON patients of all ages [4-5]. Visual improvement was noted to have a weak negative correlation with age in our patients; however, this correlation was not statistically significant. The visual improvement findings are supported by Luo et al.’s study, which reported a similar inverse relationship between visual recovery and age [7].

**Table 3:** Correlation between variables *Significant at P<0.050 BCVA: best-corrected visual acuity

Variables	Spearman’s correlation	P-value^*^
Age with initial BCVA	0.186	0.433
Age with outcome BCVA	-0.140	0.954
Age with visual improvement	-0.380	0.873
Initial BCVA with outcome BCVA	0.466	0.038

**Table 4:** Visual improvement of ON patients post-treatment a) Based on a Snellen chart ON: optic neuritis

Variables^a^	Number of patients, n=20 eyes (%)
Good (>3 lines)	10 (50.0)
Slight (1–3 lines)	4 (20.0)
Static (no improvement)	4 (20.0)
Worsening (>1 line)	2 (10.0)

The average duration of follow-up among our patients was two years and eight months. At the final review, most of the cases were idiopathic, four had infective ON, and only one case was diagnosed with seropositive NMOSD. Luo et al. proposed that poor visual recovery in their elderly population was associated with anti-aquaporin 4 seropositivity [7]. Our good visual recovery profile can be attributed to only one diagnosed case of NMOSD, and the visual outcome was also surprisingly good. Other factors determining the outcome of BCVA could include the cause of infection, as different infections had different visual outcome profiles. Infective causes detected in this study included neurosyphilis (5.9%), tuberculosis (11.8%), and toxoplasmosis (5.9%). The syphilitic ON case presented with sudden bilateral vision loss (right eye vision counting fingers and left eye vision 6/12) with bilateral swollen optic discs. After prompt treatment with intravenous penicillin G, three million units every four hours for two weeks, vision was eventually restored to 6/7.5. Although syphilitic ON patients usually present with rapid vision loss, adequate treatment can result in good visual outcomes [13]. Both of our ocular tuberculosis patients presented with poor vision (counting fingers in one patient and hand movement in the other) and did not report visual improvement despite a full course of treatment. Both patients completed two months of intensive and 10 months of maintenance anti-tuberculosis therapy. These visual outcomes were in contrast with a study by Davis et al., where almost 80% of patients with tuberculous ON achieved a final visual acuity of 6/12 or better [14]. One of the reasons for this difference may be the advanced age of presentation in our study, as the mean age of presentation for tuberculous ON was 36 years in the Davis et al. study [14]. In toxoplasmosis-related ON, the diagnosis can be made clinically and serologically with serum anti-Toxoplasma titers of IgM and IgG. Newer tools, such as polymerase chain reaction (PCR) of aqueous and vitreous samples, have higher sensitivity and specificity [15-16]. Regarding our patient, the diagnosis was made clinically with positive serological evidence of Toxoplasma IgG with no PCR of aqueous and vitreous done due to their surgically invasive nature as well as for financial reasons. Patients were treated with a six-week course of oral azithromycin and steroids. Significant visual improvement was observed after the completion of treatment.

Follow-up is crucial in ON patients due to the risk of recurrence. This is especially important in elderly patients, as this age group is usually less sensitive to sudden reduced vision as compared with younger adults. Theoretically, multiple recurrences can lead to multiple insults to the optic nerve, which may lead to optic atrophy causing moderate to severe visual loss [17]. In our study, two patients who experienced more than three episodes of recurrence had variable visual outcomes: one patient with multiple attacks throughout the follow-up period had bilateral visual deterioration and lost vision in one eye while the other patient retained relatively good vision. Neither of these patients was diagnosed with MS or NMOSD during the follow-up period.

We would like to acknowledge the limitations of this study. A confirmatory follow-up study with larger sample size is needed. The study was exploratory in nature and its aim was to obtain new data in a relatively short period of time. The limitations of a small sample size and a study conducted in one center may lead to population bias and under or overestimation of the results. With regards to ethnic profile, Malays make up 94.6% of the Kelantan population [18]; this may lead to Malays being the most presenting ethnic group with the disease. The retrospective nature of this study resulted in a non-standardized treatment course, as each patient was treated according to their etiology, with follow-up being conducted on a case-by-case basis.

## Conclusions

ON in our middle-aged and elderly study populations has no gender predilection and is most possibly idiopathic; however, demyelinating diseases and infective ON must still be ruled out in these cases. A further elaborate study is needed to support our findings. Overall visual outcomes for this age group were satisfactory as the majority of the eyes affected had slight and good visual improvements. Associated factors determining the visual outcome included the presenting BCVA and the type of infection. Among the infective causes, ocular tuberculosis caused poorer visual recovery as compared with syphilitic and toxoplasmosis-related ON cases.
